# Dynamics in ownership, access and use of long-lasting insecticidal nets in Togo: Evidence from three population-based surveys

**DOI:** 10.1371/journal.pgph.0004393

**Published:** 2025-04-02

**Authors:** Gountante Kombate, M’belou Mazimna, Kamba Andre-Marie Soubeiga, Diederick E. Grobbee, Marianne A.B. van der Sande

**Affiliations:** 1 Research and Planning Department, Ministry of Health and Public Hygiene, Lomé, Togo; 2 University Medical Centre Utrecht, Utrecht University, Utrecht, The Netherlands; 3 Department of Public Health, Institute of Tropical MedicineAntwerp, Belgium; 4 Interdisciplinary Research Laboratory in Social and Health Sciences University Joseph Ki-Zerbo, Ouagadougou, Burkina Faso; Monash University Indonesia, INDONESIA

## Abstract

Malaria remains a major public health problem in many countries in Sub Saharan Africa, including Togo, particularly among children under 5 years of age. Therefore, several mass distribution campaigns of long-lasting insecticide-treated bed nets (LLINs), which constitute an essential preventive strategy, have been conducted. The aim of this study is to assess progress in terms of equity of ownership, access and use of LLINs in a context of universal coverage among households in Togo.Data from the Togo Multiple Indicator Cluster Survey (TMICS) of 2010, the Togo Demographic and Health Survey (TDHS) of 2013-2014, and the Togo Malaria Indicator Survey (TMIS) of 2017 were used. For each survey, three main LLIN indicators were calculated: ownership (defined as % of households owning at least one LLIN), access (defined as % of households owning at least one LLIN per two people), and use (defined as use in the night before the survey by any household member). Trends from 2010 to 2017 were assessed by calculating the percentage point change between 2010 and 2017. A multivariate analysis was performed to identify factors associated with the use of LLINs in under five children. Nationally, between 2010 and 2017, LLIN ownership increased from 56.0% [54.4-58.2] to 85.0% [84.1-86.0]. LLIN access increased from 28.3% [27.0-29.2] to 71.0% [70.1-73.1], with little heterogeneity between regions. LLIN use increased from 37.1% [36.2-38.6] to 63.0% [62.5-64.7] in the whole population, with a similar trend observed among under five children. Region and type of housing showed a significant association with the use of LLINs in under five children. Considerable progress with regard to ownership, access and use of LLINs between 2010 and 2017 was observed in Togo, although LLIN coverage remained below the national targets of 100% for ownership and access for each member and 80% for use. The reduced inequity suggests efforts were well targeted to those most in need. These results can support both future policy decisions and downstream analyses of malaria prevention.

## Background

Malaria is a major global public-health problem specially in sub-Saharan Africa. The World Health Organization (WHO) recommends vector control (i.e., reducing the chances of mosquitoes biting human beings) and chemoprophylaxis (i.e., providing drugs that suppress infections) in specific population subgroups (i.e., pregnant women, children and other high-risk groups) to control the disease [1,2]. While several interventions can reduce the incidence of malaria, the use of Long-Lasting Insecticide-treated Nets (LLINs) remains a key intervention [3,4].

Togo, a country in West Africa, is also endemic for malaria, with children under 5 years of age particularly vulnerable. Togo’s population was estimated at 8,095,498 in 2022, compared with 6,191,155 in 2010 [5] The majority (57%) of the population is rural. In 2022, Togo recorded 2,224,558 cases of malaria, with more than a third of these cases among children under 5. This number of cases represents around 0.94% of the total number of malaria cases in sub-Saharan Africa, which was around 233 million in that same year [6]. Between 2010 and 2017, Togo saw notable fluctuations in the growth of its gross domestic product (GDP). The GDP growth rate remained strong, fluctuating between 4.35% and 6.54% per year over the same period [7].

Togo has committed to several international initiatives, including the Abuja Declaration of 2000, which aimed to reduce malaria mortality by 50% by 2010. This led to the development of four strategic plans to combat malaria, covering the periods 2001-2005, 2006-2010, 2011-2016 and 2017-2022 [8]. Interventions related to LLINs included: 1) free distribution through national campaigns, 2) free distribution as part of routine antenatal care and expanded immunization programs in all facilities and 3) sale by the private sector [8]. The first national LLIN distribution campaign took place in 2004. This campaign was coupled with the administration of Mebendazole tablets and the vaccination of under five children against polio [9,10]. A second national campaign to distribute LLINs, combined with vitamin A supplementation and Albendazole deworming, took place in 2008 [11,12]. Further distribution campaigns followed in 2011, 2014, 2017 and 2020 [8]. Following these campaigns, the two main indicators to assess LLIN coverage and usage were: the “percentage of households owning at least one LLIN” and the “percentage of any household member who slept under an LLIN the previous night” [13]. Consistently, these indicators revealed a significant gap between ownership and the actual use of bed nets by vulnerable groups. However, these two indicators did not provide information on whether a household has enough bed nets for all its members and whether each member has access to a bed net [14,15]. This is why a new indicator was introduced to assess access: percentage of households with at least one bed net for every two persons [16]. These three indicators allow estimation of progress and gaps.

There are many factors affecting LLIN use among those who own a LLIN. Several studies cite physical discomfort due to heat when sleeping under LLINs [14], low mosquito density in the bedroom [17], inadequate or non-existent education campaigns after LLIN distribution [11,18], and individual level of education and knowledge about the benefits of using LLINs [19]. However, while behavioural factors play a key role in failure of malaria vector control [20], the primary reason for non-use of LLIN is lack of access [14,18]; indeed, a study using population data from 25 sub-Saharan African countries [21] suggested that improving access to LLINs should be the main priority for malaria elimination.

The aim of the study was to determine whether and to what extent progress has been made towards achieving national targets, and to identify factors associated with the use of LLINs, particularly among under five children.

## Methods

We examined freely available national datasets to analyze trends in LLIN ownership, access, and use in Togo over an 8-year period, both at the national and regional levels. We calculated these indicators from the 2010 Togo Multiple Indicator Cluster Survey (TMICS), the 2013-2014 Togo Demographic and Health Survey (TDHS), and the 2017 Togo Malaria Indicator Survey (TMIS) (Table 1). Indicators were developed by the Roll Back Malaria (RBM) initiative and the Monitoring and Evaluation Reference Group (MERG) [16].

**Table 1:** Bed nets ownership, access and use indicators in all 3 surveys as defined by RBM/MERG.

Indicators	Numerator	Denominator	Gaps per indicator
**Ownership**			
Percentage of households in the study with at least one LLIN **(P1)**	Number of households owning at least one LLIN	Number of households in the survey	Number of households without at least one LLIN is calculated as **1- P1**
**Access**			
Percentage of population in the household with sufficient access to LLINs **(P2)**	Number of households owning at least one LLIN for every two household members	Number of households in the survey owning at least one LLIN	People that did not have access to LLINs [despite possessing at least 1 LLIN is calculated as **1-P2**
**Use**			
Percentage of household members who slept under an LLIN the previous night among those with access **(P3)**	Number of household members who slept under an LLIN the night before the survey	Total number of people with access to an LLIN, calculated as the sum of all access	People that did not use LLINs the previous night despite having access is calculated as **1-P3.**

### Data sources

This is a secondary analysis of data from the three population-based and nationally representative surveys mentioned above. TMICS 2010 was conducted by governmental organizations with the support and assistance of UNICEF to fill data gaps to monitor the well-being of children and women in Togo. The 2013–2014 TDHS included information on socio-economic, demographic and health indicators, while the 2017 TMIS provided a database for assessing the impact of the various strategies implemented to control malaria in Togo. All these surveys used a two-stage cluster sampling method. Using information from Togo’s last general population census in 2010, each region was subdivided into enumeration areas (EAs) in the form of villages, neighbourhoods and cities [19,20,21]. In the first stage, the EAs were drawn proportional to size and in the second stage, households were drawn randomly in each EAs selected, In the second stage, households were selected from the EAs included to form the study sample.

#### Data from Togo multiple indicator cluster surveys (TMICS) 2010.

TMICS 2010 data were collected from September 06 to December 04, 2010. In total, about 465 EAs were selected covering 6,975 households [22]. A total of 6376 women aged 15 to 49 were interviewed and 4908 under five children were included in the study.

#### Data from Togo demographic and health survey (TDHS) 2013–2014.

The TDHS 2013-2014 data were collected from November 10, 2013 to April 15, 2014. This included a representative probability sample of 9,899 households (3,840 in urban areas in 128 clusters and 6,059 in rural areas in 202 clusters). A total of 9,480 women aged 15–49 (3,591 in urban areas and 5,886 in rural areas) and 6,529 children aged 0-5 years were included in the survey [23].

#### Data from Togo malaria indicators survey (TMIS) 2017.

The 2017 TMIS data were collected from September 16 to November 17, 2017. In total, the sample consisted of 171 EAs, 5,130 households (1,800 in urban areas and 3,330 in rural areas), 4,895 women from 15 to 49 years old (1684 in urban areas and 3,211 in rural areas), and 3,271 under five children (2,441 in rural areas and 830 in urban areas) [24].

### Variables/Indicators

We calculated the three indicators mentioned above. The national target is to have 100% coverage (0% gap) for ownership and access, with a target of 80% use among high-risk populations (under five children, pregnant women and populations in targeted high-transmission areas) [8,25], thus no more than 20% gap for use.

### Ethics approval and consent to participate

For this study, ethics approval was not sought since our analysis was based on publicly available data. However, DHS reports that informed consent, both written and verbal, was obtained from all participants by the Institutional Review Board of ICF International and the Bioethics Committee for Health Research (BCRS) of Togo. The data set and permission to conduct secondary data analysis were granted by the DHS program and UNICEF.

### Statistical analysis

For data analysis, point estimates (in percentages) and 95% confidence intervals were calculated for each indicator. In addition, trend to assess the significance of the increase in LLIN ownership, access and use across the three time points (2010, 2013–2014, and 2017) and the percentage point changes between the baseline (2010) and the endline (2017) were calculated using a Chi-square test for each indicator, and statistical significance was evaluated at the 5% level. We included sampling weights in our analysis to ensure the accuracy of our estimates and to account for potential biases arising from the survey design, including stratification, clustering, and unequal probabilities of selection. These weights, which are pre-included in the DHS dataset and typically labelled as v005, v021, and v022 (depending on the dataset), are crucial for generating accurate population-level estimates. Changes by region and socio-demographic factors for each indicator between 2010 and 2017 were explored using the difference between weighted proportions. For the use indicator, considered a key public health variable, we presented the results for the whole population, as well as for under five children, who are particularly vulnerable to malaria. To assess independent associations between LLIN use and other variables, we conducted multivariate analyses using Generalized Linear Models (GLM). For these analyses, we used the Binomial family with a logit link function to estimate adjusted odds ratios. We incorporated sampling weights (v005, v021, and v022) from the DHS dataset to account for the complex survey design, including stratification, clustering, and unequal selection probabilities. The GLM was run separately for each survey year (2010, 2014, and 2017) to examine temporal trends in LLIN use. Data were analyzed using R version 4.3.3. Shapefiles of Togo’s health regions extracted from Open Street Map websites [26] were used to generate maps using QGIS 3.36.

## Results

### Global changes in LLIN ownership, access and use gaps from 2010 to 2017

**Fig 1:**
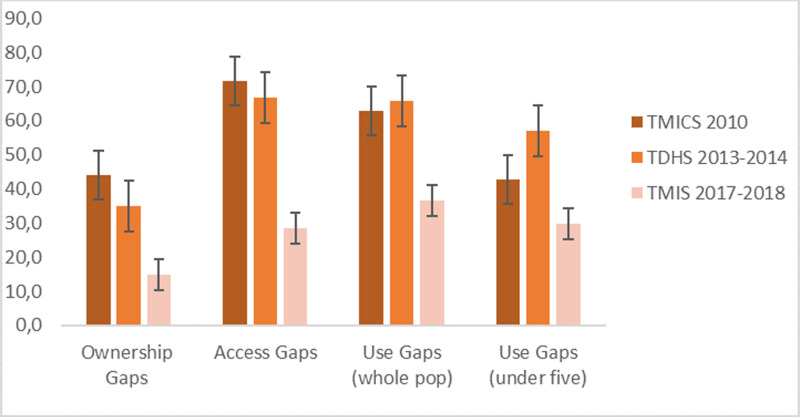
Global ownership, access and use gap.

For all three indicators (ownership, access and use) the gaps have decreased (2010–2017), with limited or no progress between 2010 and 2014, and more between 2014 and 2017 (Fig 1 and S1 Table).

### Changes in LLIN ownership from 2010 to 2017 by selected characteristics

**Fig 2:**
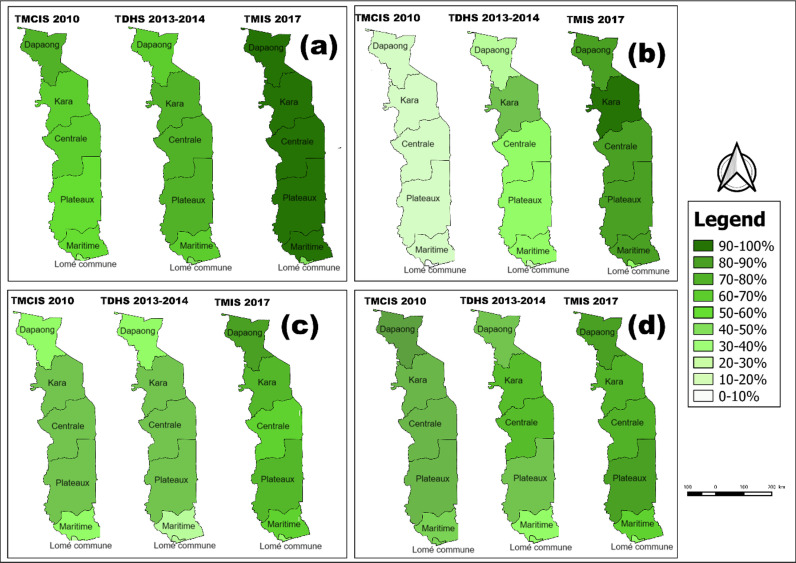
Trends in ownership, access and use of LLINs in Togo by region. Percentage of households owning at least one LLIN **(a)**, at least one LLIN for every two persons **(b)**, percentage of household population having slept under an LLIN during the night before the survey **(c)** and percentage of under five children having slept under an LLIN during the night before the survey **(d)**. Contains information from OpenStreetMap and OpenStreetMap Foundation, which is made available under the Open Database License.

LLIN ownership at the household level was 56% in 2010, 65% in 2013–2014, and 85% in 2017, showing a statistically significant increase across these three time points (p < 0.001). In rural areas, the percentage point change (2010–2017) was 35% versus 22% in urban areas (Table 2). LLIN ownership increased significantly in all household wealth quintiles from 2010 to 2017. Household ownership of at least one LLIN reached 99% in the poorest households versus 66% in the richest households (Table 2). Fig 2 shows the evolution of ownership at regional level.

**Table 2:** Changes in net ownership from 2010 to 2017 by selected characteristics.

Characteristic	TMCIS 2010		TDHS 2013-2014		TMIS 2017		Percentage point change (2010–2017)
	% (95% CI)	N	% (95% CI)	N	% (95% CI)	N	
***Residence***							
Urban	49.2 [47.2 -51.1]	2545	58.4 [56.9-59.9]	4205	71.3 [53.7-57.4]	2129	22.1 [18.3-26.4]
Rural	61.0 [59.3-62.6]	3494	70.8 [69.5-72.0]	5344	95.9 [95.1-96.6]	2780	34.9 [31.1-38.2]
***Region***							
Lome commune	41.9 [38.7-45.0]	928	51.4 [49.5-53.2]	2715	57.8 [55.1-60.4]	1334	15.9 [12.3-19.4]
Maritime	50.0 [47.7-52.2]	1931	63.8 [61.6-65.9]	1928	90.7 [88.8-92.5]	946	40.7 [37.6-43.7]
Plateaux	56.9 [54.2-59.4]	1403	72.1 [70.2-73.9]	2226	96.7 [95.6-97.6]	1292	39.8 [31.2-49.1]
Central	62.9 [58.5-67.1]	484	76.6 [73.6-79.4]	815	96.6 [94.4-98.3]	418	33.7 [23.5-45.7]
Kara	65.5 [61.9-69.0]	681	77.4 [74.8-79.9]	1041	96.9 [95.4-98.4]	524	31.4 [24.6-39.3]
Savanes	79.6 [76.3-82.7]	612	69.4 [66.3-72.5]	824	99.0 [98.3-99.9]	395	19.4 [14.4-25.2]
***Household wealth quintiles***							
Poorest	55.8 [52.9-58.7]	1120	75.5 [73.0-77.8]	1206	99.2 [98.4-99.7]	806	43.4 [38.1-48.9]
Poorer	62.6 [59.7-65.4]	1106	74.6 [72.4-76.6]	1648	98.9 [98.1-99.5]	882	36.3 [30.2-42.8]
Average	59.5 [56.6-62.3]	1121	66.5 [64.5-68.4]	2187	94.4 [92.9-95.7]	1064	34.9 [22.5-47.3]
Richer	50.6 [47.9-53.2]	1371	59.1 [57.0-61.0]	2343	73.8 [71.2-76.4]	1085	23.2 [18.7-28.3]
Richest	53.4 [50.8-56.0]	1321	58.4 [56.3-60.4]	2164	65.9 [63.0-68.7]	1072	12.5 [89.5-17.2]
***Size of the Household***							
Small (1-4 members)	59.1 [57.2-60.8]	2830	68.1 [66.8-69.3]	4992	92.3 [91.2-93.3]	2605	33.2 [27.3-39.6]
Large (5+ members)	43.4 [41.6-45.1]	3209	46.4 [44.9-47.8]	4557	74.4 [72.6-76.1]	2304	31.0 [28.2-34.1]
***House type*** ^a^							
Traditional	47.3 [45.4-48.9]	2889	45.2 [49.5-56.2]	3433	40.1 [38.7-42.2]	1224	-07.3 [06.2-09.0]
Modern	50.0 [49.0-52.8]	3150	56.2 [55.3-58.1]	6116	60.1 [59.1-62.2]	1685	10.1 [09.3-12.1]

CI: Confidence Intervals, TMICS: Togo Multiple Cluster Indicator Survey, TDHS Togo Demographic and Health, TMIS: Malaria Indicator Survey.

^a^Modern houses were defined as those with a cement, wood or metal wall, tiled or metal roof and closed eaves; all other houses were defined as traditional.

### Changes in LLIN access (1 LLIN per 2 persons) from 2010 to 2017 by selected characteristics

In all three surveys (Table 3), a higher percentage of households in rural than in urban areas were found to have access to at least one LLIN per two people. Households with 1–4 members were reported to have more often a LLIN for every 2 people than households with at least 5 members. Household members from the poorest wealth quintile experienced a percentage point change of 74.6%, which was significantly higher than the 33.8% observed in households from the richest wealth quintile.

**Table 3:** Changes in LLIN access (at least one LLIN for every two members) from 2010 to 2017.

Characteristic	TMCIS 2010		TDHS 2013-2014		TMIS 2017		Percentage point change (2010–2017)
	% (95% CI)	N	% (95% CI)	N	% (95% CI)	N	
***Residence***							
Urban	11.9 [10.6-13.1]	2545	31.8 [30.3-33.1]	4305	51.4 [49.2-53.5]	2113	39.4 [34.9 -44.1]
Rural	16.4 [15.1-17.6]	3494	33.7 [32.4-34.9]	5244	86.7 [85.4-87.9]	2769	70.3 [66.8 -73.5]
***Region***							
Lome commune	08.9 [07.1-10.6]	928	27.1 [25.4-28.7]	2715	32.5 [30.0-35.0]	1325	23.6 [19.9-28.0]
Maritime	16.3 [14.6-17.9]	1931	33.7 [31.6-35.8]	1928	83.6 [81.1-85.9]	941	67.3 [64.3-70.1]
Plateaux	14.3 [12.4-16.0]	1403	35.5 [33.5-37.4]	2226	88.3 [86.4-90.0]	1284	74.1 [65.4-81.0]
Central	13.9 [10.7-16.9]	484	37.1 [33.7-40.3]	815	85.2 [81.8-88.6]	413	71.3 [59.9-80.5]
Kara	15.6 [12.8-18.2]	681	43.9 [40.7-46.8]	1041	90.3 [87.7-92.8]	524	74.7 [67.3-80.8]
Savanes	14.9 [12.0-17.6]	612	24.7 [21.6-27.5]	824	86.8 [83.2-89.9]	395	71.9 [65.5-77.4]
***Household wealth quintiles***							
Poorest	14.2 [12.1-16.2]	1120	27.2 [24.6-29.6]	1207	88.8 [86.6-90.9]	803	74.6 [69.5-79.1]
Poorer	16.2 [14.0-18.3]	1106	34.9 [32.5-37.1]	1648	90.1 [88.0-91.9]	880	73.9 [67.8-79.1]
Average	12.1 [10.1-13.9]	1121	36.3 [34.2-38.3]	2187	86.1 [83.9-88.1]	1056	74.2 [62.2-83.1]
Richer	10.8 [09.1-12.4]	1371	31.7 [29.8-33.6]	2343	55.7 [52.7-58.6]	1081	44.9 [39.3-50.5]
Richest	10.9 [09.2-12.5]	1321	32.5 [30.5-34.4]	2164	44.7 [41.6-47.6]	1069	33.8 [28.2-39.8]
***Size of the Household***							
Small (1-4 members)	35.3 [33.5-37.1]	2830	34.4 [33.1-35.7]	4992	88.2 [86.9-89.4]	2605	52.9 [46.3-59.3]
Medium (5+ members)	26.8 [25.2-28.3]	3209	29.1 [27.7-30.4]	4557	69.3 [67.3-71.1]	2304	42.5 [39.3-45.7]
***House type*** ^a^							
Traditional	47.1 [46.5-48.9]	2623	43.0 [42.5-44.2]	3640	42.1 [39.5-44.0]	1657	-05.0 [05.1-09.5]
Modern	51.5 [49.1-54.4]	3416	55.2 [54.3-56.1]	5909	58.0 [57.2-60.1]	3225	06.5 [13.3-37.1]

CI: Confidence Intervals, TMICS: Togo Multiple Cluster Indicator Survey, TDHS Togo Demographic and Health, TMIS: Togo Malaria Indicator Survey.

^a^Modern houses were defined as those with a cement, wood or metal wall, tiled or metal roof and closed eaves; all other houses were defined as traditional

### Changes in use of LLINs among household members from 2010 to 2017 by selected characteristics

From 2010 to 2017, a greater increase in LLIN use was observed in rural areas, rising from 49.1% to 93.6%, compared to urban areas, where the increase was from 39.9% to 64.0%. Household members from the poorest wealth quintile showed a greater increase in LLIN use, with a percentage point change of 50.9%, compared to 17.8% in households from the richest wealth quintile (Table 4).

**Table 4:** Changes in LLIN use in whole population from 2010 to 2017.

Variables	TMCIS 2010		TDHS 2013-2014		TMIS 2017		Percentage point change (2010–2017)
	% (95% CI)	N	% (95% CI)	N	% (95% CI)	N	
***Residence***							
Urban	39.9 [39.1-40.7]	12972	43.8 [43.1-44.5]	20488	64.0 [62.9-65.0]	8147	24.3 [22.8-25.3]
Rural	49.1 [48.2-49.8]	15966	51.8 [51.1-52.4]	22488	93.6 [93.1-94.0]	13275	44.5 [42.6-46.3]
***Region***							
Lome commune	32.6 [31.1-34.2]	3538	38.8 [37.6-40.0]	6162	46.3 [44.9-47.6]	5067	13.6 [12.1-15.5]
Maritime	43.6 [42.1-45.0]	4724	47.8 [46.6-48.9]	7182	87.7 [86.6-88.7]	4048	44.1 [40.4-47.8]
Plateaux	45.4 [44.0-46.8]	4922	51.8 [50.6-52.9]	7362	95.4 [94.8-95.9]	5486	49.9 [45.8-54.1]
Central	51.4 [50.4-53.1]	5614	56.8 [55.6-57.8]	7462	93.8 [92.7-94.7]	2165	42.0 [40.3-43.6]
Kara	51.8 [50.5-53.1]	5615	59.8 [58.6-60.8]	7662	96.2 [95.4-96.9]	2363	44.4 [42.6-46.1]
Savanes	41.8 [40.3-43.2]	4526	46.8 [45.6-47.9]	7146	96.1 [95.2-96.8]	2293	54.3 [52.2-56.3]
***Household wealth quintiles***							
Poorest	45.4 [44.1-46.6]	5930	49.8 [48.7-50.8]	8665	96.4 [95.8-96.9]	4288	50.9 [48.5-53.4]
Poorer	49.1 [47.8-50.2]	6411	53.8 [52.7-54.8]	8810	96.5 [95.9-97.0]	4280	47.4 [45.2-49.5]
Average	46.3 [45.0-47.5]	6046	50.8 [49.7-51.8]	8705	92.8}92.0-93.5]	4319	46.4 [44.1-48.8]
Richer	39.9 [38.6-41.2]	5210	43.8 [42.7-44.8]	8395	67.1 [65.6-68.5]	4264	27.1 [24.4-30.1]
Richest	40.9 [39.5-42.2]	5341	44.8 [43.7-45.8]	8401	58.7 [57.2-60.2]	4271	17.8 [15.6-20.2]
***Size of the Household***							
1-4 members	39.9 [38.9-40.8]	11320	42.3 [41.6-42.9]	20056	78.8 [78.0-79.5]	10198	38.9 [36.1-41.1]
5+ members	49.0 [48.2-49.7]	17618	51.0 [50.3-51.6]	22920	88.1 [87.5-88.7]	11224	39.1 [37.9-40.3]
***House type*** ^a^							
Traditional	50.1 [49.0-52.4]	2623	46.0 [44.5-47.2]	3322	60.1 [58.0-62.4]	1532	10.0 [09.1-13.5]
Modern	52.0 [51.0-54.8]	3250	55.2 [54.3-56.1]	4760	61.1 [59.2-62.9]	2236	09.1 [07.3-11.1]

CI: Confidence Intervals, TMICS: Togo Multiple Cluster Indicator Survey, TDHS Togo Demographic and Health, TMIS: Togo Malaria Indicator Survey.

^a^Modern houses were defined as those with a cement, wood or metal wall, tiled or metal roof and closed eaves; all other houses were defined as traditional

### Changes in LLIN use among under five children from 2010 to 2017 by selected characteristics

Among under five children, use of LLINs in the night preceding each survey increased from 57% in 2010 to 70% in 2017 (p <0.001). In rural areas, children’s use of LLINs was higher (Table 5). There was a percentage point change of 17.9% in rural versus 3.4% in urban areas. Children from the poorest households had a higher percentage point change (23.7%) in use of LLINs, compared with children from the richest households (1.5%) (Table 5).

**Table 5:** Changes in use of LLINs among under five children.

**Characteristic**	TMCIS 2010		TDHS 2013-2014		TMIS 2017-2017		Percentage point change (2010–2017)
	% (95% CI)	N	% (95% CI)	N	% (95% CI)	N	
***Residence***							
Urban	50.7 [48.1-53.2]	1532	37.8 [35.8-39.7]	2290	54.1 [51.2-56.9]	1173	03.4 [29.2-39.2]
Rural	60.1 [58.3-61.7]	3214	45.3 [43.8-46.7]	4332	78.0 [76.2-79.6]	2219	17.9 [15.4-20.4]
***Region***							
Lome commune	48.1 [43.7-52.3]	512	34.6 [32.1-37.0]	1480	43.0 [39.3-46.4]	755	05.1 [44.5-57.2]
Maritime	48.1 [45.4-50.7]	1347	35.9 [33.2-38.6]	1203	64.5 [60.5-68.2]	596	16.4 [13.9-19.2]
Plateaux	59.8 [56.8-62.7]	1055	46.0 [43.5-48.4]	1598	82.4 [79.8-84.9]	845	22.6 [17.4-28.7]
Central	59.1 [54.5-63.8]	443	52.4 [48.4-56.1]	654	70.0 [65.8-75.6]	332	11.9 [07.7-19.2]
Kara	59.0 [54.5-63.7]	641	50.5 [47.0-53.9]	790	79.7 [75.6-83.7]	379	20.7 [16.2-26.1]
Savanes	72.9 [69.6-76.0]	748	45.3 [42.8-49.4]	897	87.0 [83.9-89.9]	484	14.1 [10.4-18.8]
***Household wealth quintiles***							
Poorest	55.3 [52.3-58.2]	1114	44.7 [42.1-47.2]	1432	79.0 [76.1-81.8]	790	23.7 [19.3-28.6]
Poorer	63.2 [60.2-66.1]	1043	46.3 [43.6-48.9]	1384	85.2 [82.5-87.8]	716	22.0 [17.8-26.8]
Average	62.4 [59.2-65.4]	941	43.9 [41.2-46.5]	1348	74.3 [70.8-77.6]	640	11.9 [08.7-16.1]
Richer	49.5 [46.1-52.8]	851	38.7 [35.9-41.3]	1257	53.3 [49.3-57.1]	639	03.8 [01.7-04.6]
Richest	53.3 [49.8-56.7]	797	39.1 [36.2-41.8]	1201	51.8 [47.7-55.7]	607	01.5 [00.9-0.2.1]
***Size of the Household***							
Small (1-4 members)	52.5 [50.0-54.8]	1639	39.4 [37.4-41.2]	2489	63.3 [60.7-65.7]	1404	10.8 [07.4-15.4]
Medium (5+ members)	68.2 [66.5-69.8]	3107	57.3 [55.7-58.8]	4133	76.0 [74.1-77.8]	1988	07.8 [75.4-80.3]
***House type*** ^a^							
Traditional	48.3 [46.5-49.9]	2240	45.0 [48.5-57.2]	2701	42.1 [39.5-44.0]	1010	-06.2 [05.8-08.7]
Modern	51.5 [49.1-55.8]	2506	55.2 [54.3-58.1]	3921	59.1 [57.2-62.3]	2382	07.9 [06.5-09.4]

CI: Confidence Intervals, TMICS: Togo Multiple Cluster Indicator Survey, TDHS: Togo Demographic and Health, TMIS: Togo Malaria Indicator Survey.

^a^Modern houses were defined as those with a cement, wood or metal wall, tiled or metal roof and closed eaves; all other houses were defined as traditional.

### Multivariate analysis of LLIN use among under five children from 2010 to 2017

The Table 6 presents the results of the multivariate analysis of LLIN use among under five children. No independent significant association was found between LLIN use and rural/urban residence during the study period. Also, no such association was observed between LLIN use and household wealth quintile. However, there was an association between LLIN use and region upon adjustment for wealth, household size, and urbanisation. Children living in the Plateaux region were twice as likely in 2010 and 2017 to be using LLINs than those in the Lomé commune. Also, in 2017, adjusted for the same factors as in 2010, children living in modern houses were twice as likely to use LLINs than those living in traditional houses; such an independent association was not observed in 2010 and 2014.

**Table 6:** Factors associated with LLIN use among under five children from 2010 to 2017.

Characteristic	TMCIS 2010		TDHS 2013-2014		TMIS 2017	
	OR [95% CI]	a.OR [95% CI]	OR [95% CI]	a.OR [95% CI]	OR [95% CI]	a.OR [95% CI]
***Residence***						
Urban	Ref.	Ref.	Ref.	Ref.	Ref.	Ref.
Rural	1.3 [1.1 - 1.4]	1.1 [0.7 - 1.3]	1.2 [1.0 - 1.3]	1.0 [0.7 - 1.3]	1.6 [1.2 - 1.7]	1.1 [0.6 - 1.4]
***Region***						
Lomé commune	Ref.	Ref.	Ref.	Ref.	Ref.	Ref.
Maritime	1.2 [1.1 - 1.3]	0.9 [0.8 - 1.2]	1.2 [1.0 - 1.3]	0.8 [1.6 - 1.0]	1.8 [1.7 -2.0]	**1.2 [1.1 - 1.6]**
Plateaux	2.1 [1.9 - 2.3]	**2.0 [1.7 - 2.2]**	1.7 [1.6 - 1.8]	**1.5 [1.2 - 1.7]**	2.3 [2.2 -2.5]	**2.0 [1.6 - 2.3]**
Central	1.7 [1.6 - 1.9]	1.3 [0.9 - 1.5]	1.8 [1.4 - 1.9]	**1.4 [1.2 - 1.8]**	2.0 [1.8 -2.2]	**2.1 [1.8 - 2.3]**
Kara	1.8 [1.7 - 2.0]	**1.9 [1.7 - 2.1]**	1.8 [1.7 - 1.9]	**1.3 [1.1 - 1.5]**	2.2 [2.0 - 2.4]	**1.6 [1.2 - 1.9]**
Savanes	1.4 [1.2 - 1.6]	**1.3 [1.1 - 1.5]**	1.3 [1.2 - 1.5]	0.7 [0.5 - 1.0]	2.5 [2.3 - 2.7]	**2.1 [1.9 - 2.4]**
***Household wealth quintiles***						
Poorest	Ref.	Ref.	Ref.	Ref.	Ref.	Ref.
Poorer	1.1 [1.0 - 1.2]	1.0 [0.7 - 1.2]	1.1[0.8 - 1.2]	0.9 [0.9 -1.2]	1.1[1.0 - 1.2]	0.9 [0.8 - 1.1]
Average	0.9 [0.7 - 1.1]	0.8 [0.6 - 1.0]	0.9 [0.9 -1.0]	0.7 [0.6 - 0.9]	0.8 [0.7 - 0.9]	0.7 [0.6 - 0.9]
Richer	0.8 [0.6 - 0.9]	0.6 [0.4 - 0.8]	0.8 [0.6 - 0.9]	0.6 [0.4 - 0.7]	0.5 [0.4 - 0.6]	0.4 [0.3 - 0.5]
Richest	0.7 [0.5 - 0.8]	0.4 [0.2 - 0.6]	0.7 [0.6 - 0.8]	0.5 [0.3 - 0.7]	0.4 [0.3 - 0.6]	0.3 [0.2 - 0.4]
***Size of the Household***						
1-4 members	Ref.	Ref.	Ref.	Ref.	Ref.	Ref.
5+ members	1.2 [1.0 - 1.5]	0.8 [0.7 - 1.0]	1.1 [0.9 - 1.4]	0.9 [0.8 - 1.0]	1.0 [08 - 1.3]	0.7 [0.4 - 1.2]
***House type*** ^a^						
Traditional	Ref.	Ref.	Ref.	Ref.	Ref.	
Modern	1.3 [1.2- 1.4]	1.0 [0.9 - 1.3]	1.5 [1.4 - 1.6]	1.1 [0.8 - 1.4]	3.3 [3.0 - 3.6]	**1.9 [1.3 - 2.5]**

OR: crude Odd ratio; a.OR: Adjusted odd ratio for all other factors considered in the multivariate analysis; CI: Confidence Intervals; TMICS: Togo Multiple Cluster Indicator Survey; TDHS Togo Demographic and Health; TMIS: Togo Malaria Indicator Survey.

^a^Modern houses were defined as those with a cement, wood or metal wall, tiled or metal roof and closed eaves; all other houses were defined as traditional.

## Discussion

This study assessed trends in gaps between household people ownership, access and use of LLINs in Togo according to the indicators recommended by the MERG and explored risk factors associated with the use of LLINs in under five children.

There was a significant improvement in all three indicators from 2010 to 2017 aligning with regional trends toward higher ownership, access and utilization in Sub-Saharan Africa [27]. During this period, Togo experienced 3 LLIN mass distribution campaigns. Significant improvements were observed among households in the poorest wealth quintile. This unexpected trend is a challenge to the assumption that increased LLIN coverage leads to decreased health inequalities [28]. Further investigation is needed to understand these dynamics and their implications for equitable health interventions.

This process showed slow progress initially while progress accelerated later on. The limited improvement in malaria metrics between 2010 and 2013–2014 in Togo was likely due to implementation challenges, variations in funding, and shifts in policy priorities. LLINs may have failed to be distributed and used effectively due to logistical constraints, supply chain problems, and inadequate community engagement [29]. Moreover, changes in funding from both domestic and international sources could have had an impact on the resources available for LLIN campaigns and distribution efforts[29–31]. Despite progress in household ownership of LLINs, about a third of the household population still did not have access to an LLIN. This hinders universal use and the achievement of national targets [28]. Access may be determined by a number of factors not assessed here [32], including household composition (e.g., number of children), sleeping spaces and other household arrangements [33,34]. These factors and others need to be explored in more in-depth studies, as the current nationwide surveys did not provide such data. The relatively lower changes in LLIN ownership, use, and access in Lomé commune could be attributed to its urban setting, where alternative preventive measures such as insecticide sprays or better housing structures might reduce reliance on LLINs [35,36]. We observed no significant association between household wealth quintile and LLIN use in under five children. The same results were found in Gabon [37]. Higher-income households may have greater access to other malaria prevention methods, further contributing to the reduced reliance on LLINs in comparison to lower-income households. Although household wealth quintile has been shown to influence household LLIN ownership in some contexts [21,38], it had no influence on their use after ownership. Since LLIN were distributed free of charge in Togo, it is less likely that LLIN ownership (and therefore LLIN use) depended on household wealth quintile.

The results also showed that rural households had better access to LLINs than urban households throughout the study period and achieved the highest percentage of use. The same pattern has been observed in Ghana [39]. This likely reflects the impact of the Ministry of Health’s LLIN distribution campaigns, which prioritized rural areas to mitigate higher malaria transmission risks [40]. In contrast, our multivariate analyses showed no significant association between place of residence and use of LLINs in under five children. This corroborates the results found in rural Cameroon [41] where the LLINs were not hung over the bed, but were spread over the mattresses to kill the insects. This attitude points to a misplaced appropriation of the LLIN, used for something other than the purpose for which it was created [42]. This suggests that if individuals do not use LLINs properly, they may repurpose them for other purposes, possibly due to specific beliefs or misconceptions about the disease and the role of LLINs in prevention [41]. A mosquito net may not be perceived and known as a tool for protection against malaria, but rather as a means of combating the nuisance caused by mosquitoes or other insects [4]. To what extent this is also relevant in Togo remains to be explored. In the Gambia [43], a malaria control trial using LLINs showed that perceptions of malaria, its causes and means of prevention influenced attitudes towards the use of LLINs. Behavioural change actions and health education could be important to maintain malaria control efforts [42]. Another crucial aspect to emphasize is the quality of the LLINs, e.g., torn with holes, not (re)impregnated with insecticide; also, this aspect was not addressed in our nation-wide surveys. Non-use of LLINs, particularly if they are in good condition, reflects a behavioural failure, requiring targeted behavioural and education interventions [15]. The regional differences in the use of LLINs among under five children observed may be linked to cultural differences or ecological environments characterized by different transmission levels [19]. The observed decline in LLIN ownership, access, and use among under-five children, particularly in traditional house types, may stem from challenges in ensuring consistent LLIN distribution, limited reach in marginalized areas, and a potential reduction in perceived malaria risk. These factors underscore the need for more targeted and sustained intervention strategies [28].

All these results demonstrate the complexity of malaria as a disease and the challenges associated with assessing the impact of individual control efforts. Furthermore, the government of Togo has implemented several interventions across the country to control malaria, including seasonal malaria chemoprevention for children aged 3 to 59 months, indoor residual spraying, intermittent preventive treatment for pregnant women, and community and hospital-based screening and treatment [44]. These interventions likely influenced the results observed in 2014 by contributing to increased LLIN ownership, access, and use. Considerable progress has been made by the government, and extending this type of analysis to other interventions mentioned above could add value to evidence-based decision-making.

In addition, Togo’s GDP growth has shown consistent growth, particularly since 2008, with rates exceeding 5% annually until the onset of the COVID-19 pandemic [45]. This economic growth in Togo has probably played a role in improving malaria measurements and access to disease control tools. Studies have shown that per capita income rises with a decrease in malaria incidence. For instance, a 10% reduction in malaria incidence can result in a nearly 0.3% increase in per capita income, indicating that improving malaria metrics can contribute to economic growth [31,46]. Considering this factor could provide a valuable context for understanding progress in the fight against malaria[47,48].

Despite the national scale of the data, which is an important strength of this study, there are also some limitations. The data from these different surveys were collected at different seasons of the year[48]. TMIS and TMICS data were collected during the period of high malaria transmission, while TDHS data were collected at the end of the transmission period. This difference could potentially affect the results and also underestimate or overestimate the effect size, as the use of insecticide-treated nets can be seasonal depending on the perceived nuisance of mosquitoes [23,33], and may also partially explain less observed use in the second (TDHS) survey. In addition, the data are based on respondents’ self-reports during the various surveys and could therefore be biased by social desirability [49].

## Conclusion

This study, which aimed to explore the trends in household ownership, access and use of LLINs and identify potential gaps, showed that considerable progress was made in Togo between 2010 and 2017. However, there is still a long way to go to achieve the government’s target of 100% of households owning LLINs, with full access for each member, meaning 1 LLIN for 2 persons, and at least 80% pregnant women and children using them. Regional differences and health education regarding ownership, access and use should be considered when designing future malaria control programs.

## Supporting information

S1 Table Overall trends in malaria prevention indicators in Togo from 2010 to 2017.
(DOCX)
