# Supplementary material for: Dynamics in ownership, access and use of long-lasting insecticidal nets in Togo: Evidence from three population-based surveys
Source: PLOS Glob Public Health. 2025 Apr 2;5(4):e0004393. doi: 10.1371/journal.pgph.0004393 (PMC11964233; doi:10.1371/journal.pgph.0004393)
Supplement: S1 Table — Overall trends in malaria prevention indicators in Togo from 2010 to 2017. (DOCX) [file pgph.0004393.s001.docx]

S1 Table: Overall trends in malaria prevention indicators in Togo from 2010 to 2017

|  | TMICS 2010 | TDHS 2013-2014 | TMIS 2017-2018 |
| --- | --- | --- | --- |
| Owning at last 1 LLIN | 56.0 [54.4-58.2] | 65.1 [63.9-66.7] | 85.0 [84.1-86.0] |
| Access (1 LLIN for 2 persons) | 28.3 [27.0-29.2] | 33.3 [32.0-34.8] | 71.6 [70.1-73.1] |
| LLIN Use among those access (whole pop) | 37.1 [36.2-38.6] | 34.2 [33.2-35.3] | 63.3 [62.5-64.7] |
| LLIN Use among those access (under five) | 57.1 [56.3-58.0] | 43.0 [41.8-44.8] | 70.2 [68.8-72.0] |
| Ownership Gaps | **44.0 [43.1-45.0]** | **34.9 [33.6-36.8]** | **15.0 [14.1-16.3]** |
| Access Gaps | **71.7 [70.4-72.2]** | **66.7 [64.9-67.4]** | **28.4 [26.7-30.0]** |
| Use Gaps (whole pop) | **62.9 [60.8-64.1]** | **65.8 [64.2-66.9]** | **36.7 [34.1-37.2]** |
| Use Gaps (under five) | **42.9 [41.2-44.0]** | **57.0 [56.1-58.7]** | **29.8 [28.8-31.9]** |
